# Description of a nationwide structure for monitoring nosocomial outbreaks of (highly resistant) microorganisms in the Netherlands: characteristics of outbreaks in 2012–2021

**DOI:** 10.1186/s13756-023-01350-9

**Published:** 2023-12-08

**Authors:** Sjoukje HS Woudt, Annelot F Schoffelen, Florine NJ Frakking, E Ascelijn Reuland, Juliëtte A Severin, Marije den Drijver, Anja Haenen, Marga MG Nonneman, Daan W Notermans, Desiree CM aan de Stegge, Sacha F de Stoppelaar, Christina MJE Vandenbroucke-Grauls, Sabine C de Greeff

**Affiliations:** 1 Center for Infectious Disease Control, National Institute for Public Health and the Environment (RIVM), Bilthoven, The Netherlands; 2 Department of Medical Microbiology, University Medical Center Utrecht, Utrecht, The Netherlands; 3 Saltro Diagnostic Center for Primary Care, Utrecht, The Netherlands; 4 Department of Medical Microbiology and Infectious Diseases, Erasmus MC University Medical Center, Rotterdam, The Netherlands; 5 Department of Infection Control, Maasstad Hospital, Rotterdam, The Netherlands; 6 TanteLouise, Bergen op Zoom, The Netherlands; 7 Department of Medical Microbiology and Infection Prevention, Amsterdam University Medical Centers, Amsterdam, The Netherlands; 8 Beweging 3.0, Amersfoort, The Netherlands; 9 Infectious diseases department, Amsterdam Academic Medical Center, Amsterdam, The Netherlands; 10 Department of Clinical Epidemiology, Aarhus University Hospital, Aarhus, Denmark

**Keywords:** Nosocomial infections, Outbreaks, Multidrug resistance, Infection control, Epidemiology, Surveillance

## Abstract

**Background:**

Before 2012, established national surveillance systems in the Netherlands were not able to provide a timely, comprehensive epidemiological view on nosocomial outbreaks. The Healthcare-associated Infections and AntiMicrobial Resistance Monitoring Group (SO-ZI/AMR) was initiated in 2012 for timely national nosocomial outbreak monitoring and risk assessment. This paper aims to describe the achievements of the SO-ZI/AMR by presenting characteristics of outbreaks reported in 2012–2021.

**Methods:**

Hospitals and, since 2015, long-term care facilities (LTCF) were requested to report outbreaks when (1) continuity of care was threatened, or (2) transmission continued despite control measures. A multi-disciplinary expert panel (re-)assessed the public health risk of outbreaks during monthly meetings, using 5 severity phases and based on data collected via standardised questionnaires. We descriptively studied the panel’s consensus-based severity classification, distribution of (highly resistant) microorganisms, and duration and size of outbreaks between April 2012 and December 2021.

**Results:**

In total, 353 hospital outbreaks and 110 LTCF outbreaks were reported. Most outbreaks (hospitals: n = 309 (88%), LTCF: n = 103 (94%)) did not progress beyond phase 1 (no public health implications, outbreak expected to be controlled within two months), one hospital outbreak reached phase 4 (insufficient/ineffective response: possible public health threat, support offered). Highly resistant microorganisms (HRMO) were involved in 269 (76%) hospital and 103 (94%) LTCF outbreaks. Most outbreaks were caused by methicillin-resistant *Staphylococcus aureus* (MRSA; n = 93 (26%) in hospitals, n = 80 (72%) in LTCF), vancomycin-resistant *Enterococcus faecium* (VRE; n = 116 (33%) in hospitals, n = 2 (2%) in LTCF) and highly resistant *Enterobacterales* (n = 41 (12%) in hospitals, n = 20 (18%) in LTCF). Carbapenemase-producing gram-negative bacteria were involved in 32 (9.1%) hospital and five (4.5%) LTCF outbreaks. In hospitals, VRE outbreaks had the longest duration (median 2.3; range 0.0-22.8 months) and widest range of affected patients (median 9; range 2-483).

**Conclusions:**

The SO-ZI/AMR provided national insight into the characteristics of nosocomial outbreaks over the past decade. HRMO outbreaks – mostly caused by MRSA, VRE (in hospitals) and highly resistant *Enterobacterales* – occurred regularly, but most of them were controlled quickly and did not develop into a public health threat. The SO-ZI/AMR has become a solid monitoring body, essential to assess risks and raise awareness of potential HRMO threats.

**Supplementary Information:**

The online version contains supplementary material available at 10.1186/s13756-023-01350-9.

## Background

Transmission of pathogens has resulted in nosocomial outbreaks for decades [1, 2]. The Netherlands is a country with high standards of infection prevention and control and low levels of antimicrobial resistance. To monitor healthcare-associated infections and antimicrobial resistance in the country, several national surveillance systems are in place in which healthcare facilities and medical microbiology laboratories can participate on a voluntary basis. However, these systems are limited in scope (e.g. selected pathogens, selected individual laboratories, limited epidemiological information) and/or timeliness. Therefore, they may not be able to timely identify (multi-institutional) nosocomial outbreaks.

A prolonged hospital outbreak of OXA-48-producing *Enterobacterales* in 2011, affecting at least 118 patients [3], illustrated the risk of rapid spread of highly resistant microorganisms (HRMO). It demonstrated a need for early information and transparency, in order to respond quickly to possible public health threats in the future. Therefore, the minister of Health, Welfare and Sport initiated the ‘Healthcare-associated Infection and AntiMicrobial Resistance Monitoring Group (SO-ZI/AMR)’ in April 2012 [4]. The SO-ZI/AMR is a structure for outbreak monitoring and risk assessment, aiming to timely identify nosocomial outbreaks with a possible public health threat throughout the country. The SO-ZI/AMR tasks are performed by an expert panel consisting of medical microbiologists, infection prevention and control practitioners, infectious disease physicians and epidemiologists. They are representatives of the Center for Infectious Disease Control, embedded in the National Institute for Public Health and the Environment (RIVM-CIb), the Dutch Society of Medical Microbiology (NVMM) and the Association for Hygiene and Infection Control (VHIG). Since 2015, when the target group of the SO-ZI/AMR was expanded to also include long-term care facilities (LTCF, including medical rehabilitation centres), an elderly care physician of the Dutch Association of Elderly Care Physicians and Social Geriatricians (Verenso) is also a member of the expert panel. The request to notify outbreaks is also disseminated via these associations for health professionals.

Outbreaks can be reported by medical microbiologists, infectious disease specialists, infection control practitioners, or elderly care physicians, authorised by their healthcare facility to report. Data are collected using standardised questionnaires (see Additional file 1 for examples) and registered in a database maintained by the SO-ZI/AMR secretary, consisting of selected RIVM-CIb representatives. For each outbreak, the notifier can choose to disclose the identity of the institution, or report anonymously. All notifications are based on voluntarily provided information. However, in 2012, umbrella bodies representing all academic hospitals and general hospitals formally committed themselves to reporting nosocomial outbreaks to the SO-ZI/AMR. Reporting an outbreak to the SO-ZI/AMR does not replace the mandatory – under the Dutch Public Health Act – reporting of notifiable disease cases to the municipal health service (GGD).

During monthly meetings, all active outbreaks are discussed by the SO-ZI/AMR. Feedback on (updates of) the risk assessment as determined by the expert panel, is provided to notifiers after the meetings. In addition, when requested by the notifier, the SO-ZI/AMR may initiate closer collaboration between healthcare facilities in order to share experiences and provide support in outbreak control. This may include advice on diagnostic and epidemiological methods to map the outbreak – e.g. (the extensiveness of) contact investigations, data collection – and ways to implement control measures such as cleaning regimens in facilities with limited knowledge on infection control or limited capacity. An overview and update of active outbreaks is communicated to a large but defined group of infectious diseases professionals nationwide on a monthly basis, as part of a confidential report from the national early warning committee for infectious diseases [5]. A summary of all outbreaks is provided in the national annual report on antimicrobial consumption and resistance in the Netherlands (NethMap) to inform both professionals and the general public [6]. This paper aims to describe the achievements of the SO-ZI/AMR by presenting the characteristics of nosocomial outbreaks of (highly resistant) microorganisms reported to the SO-ZI/AMR between April 2012 and December 2021.

## Methods

### Notification procedure of outbreaks

The Netherlands, a country with around 17.5 million inhabitants, has 113 hospital locations, including 8 academic hospitals [7]. Furthermore, there are around 3000 locations of LTCF, e.g. nursing homes and rehabilitation centres. Since April 2012, hospitals are requested to report nosocomial outbreaks of infectious pathogens, including HRMO, to the SO-ZI/AMR. During the period of the current study, criteria to report an outbreak to the SO-ZI/AMR were: (1) an outbreak threatens healthcare continuity, for instance when wards need to be closed or when a healthcare institute stops new admissions to control the outbreak, and/or (2) transmission of the outbreak strain continues despite appropriate infection control measures. For LTCF, who were requested to report since 2015, notification criteria were stricter and included HRMO outbreaks only, although the abovementioned criteria were not a requirement for LTCF. The decision to notify the SO-ZI/AMR was based on professional judgement by the authorised representative of the healthcare institution. For definitions of HRMO, the SO-ZI/AMR referred to national guidelines for the control of HRMO, developed by the Dutch Working party on Infection Prevention (WIP) [8–10] and NVMM [11]. A cluster as confirmed by molecular typing was not a prerequisite for notification.

At notification, preliminary epidemiological characteristics of the outbreak were reported to the SO-ZI/AMR, via email or the NVMM website (for members only). Information stored in the database included notification date, type and location of the institution, department(s) involved in the outbreak, type of (highly resistant) microorganism, resistance gene (if applicable and available), reason for reporting ((threat of) ward closure, ongoing transmission, or both), request for support (yes/no), number of patients and number of healthcare workers (when relevant) involved, and types of infection prevention measures that were taken (e.g. isolation, cleaning and disinfection, ward closure).

### Classification and monitoring of outbreaks

Based on the information provided at notification, the SO-ZI/AMR assessed the risk of the outbreak for public health. To classify the severity of a possible public health threat, outbreaks were categorised in one of five phases, 1 being the lowest, and 5 the highest risk for public health (Table 1). In monthly meetings, the SO-ZI/AMR continued to assess the phase of each active outbreak based on updated information on the course of the outbreak – requested from the notifier before each monthly meeting – using a standardised follow-up questionnaire (see Additional file 1 for an example). The dates of the monthly meetings in which the different risk phases were assigned by the SO-ZI/AMR expert panel, were registered in the database. For outbreaks in phase 3 and 4, representatives of the healthcare institutes were invited to the meetings at least once to discuss the outbreak and measures taken to control it. The SO-ZI/AMR did not assess the quality or adequacy of control measures, as this is the responsibility of the Health and Youth Care Inspectorate (IGJ). Phase 0 was assigned once an outbreak was contained, i.e. no new transmissions/cases during the microorganism-specific incubation time since the last case. At that point, an end-of-outbreak questionnaire was sent to the notifier for a final update on the outbreak characteristics (Additional file 1).

**Table 1:** Phasing of outbreaks by the SO-ZI/AMR

Phase	Definition
Phase 1	No implications for public health expected, outbreak expected to be controlled soon (maximum duration 2 months).
Phase 2	Duration longer than expected (> 2 months), request information for monitoring
Phase 3	Possible threat to public health, contact and/or invite representatives of the healthcare institute for more information
Phase 4	Insufficient or ineffective response, outbreak not controlled: offer support
Phase 5	Either: offered support is refused, or despite support, control measures still insufficient or ineffective: Health and Youth Care Inspectorate is called upon

SO-ZI/AMR: Healthcare-associated Infection and AntiMicrobial Resistance Monitoring Group

### Analysis of outbreaks 2012–2021

For the current study, we examined the outbreaks reported in the first ten years of the SO-ZI/AMR (April 2012 – December 2021). Follow-up data for these outbreaks were included until March 23th 2023. We described the characteristics – request for help (yes/no), reason for reporting, highest phase assigned, microorganism involved, and resistance profile where applicable – in terms of numbers and percentages by year of notification, for hospital and LTCF outbreaks separately. Furthermore, we calculated the duration of outbreaks (median (range)) in months, the percentage of outbreaks that involved intensive care units (ICU), and the size of the outbreak in terms of patients involved (median (range), staff excluded), by healthcare setting and microorganism. These analyses by microorganism were performed regardless of resistance profile, except for *Enterobacterales*, where we distinguished extended-spectrum beta-lactamase (ESBL) producing *Enterobacterales*, carbapenemase-producing *Enterobacterales* (CPE), and all other *Enterobacterales*, including fluoroquinolone and aminoglycoside-resistant (FQAG-R) which are considered HRMO according to Dutch definitions [8]. As the start and end dates of the outbreak were often not (accurately) registered, the exact duration of the outbreak could not be determined for many outbreaks. Outbreak duration was therefore defined as the duration between the date of reporting by the healthcare institute, to the date of the SO-ZI/AMR monthly meeting in which the outbreak was assigned Phase 0. Analyses were performed using R software version 4.2.0 within Rstudio version 2022.22.2 [12].

## Results

### Characteristics of reported outbreaks 2012–2021

Between April 2012 and December 2021, 463 outbreaks were reported to the SO-ZI/AMR (Table 2). The number of outbreaks reported increased between 2012 and 2015 (from 20 to 61), was lower in 2016 (50), remained fairly stable from 2017 to 2019 (range 58–60), and was lower in 2020 in 2021 (34 and 27, respectively). 76% of outbreaks (n = 353) occurred in hospitals, and 24% (n = 110) in LTCF. Outbreaks were reported by 95 hospitals and 80 LTCF. The median number of outbreaks reported by each healthcare facility was 3 (range 1–23) for hospitals and 1 (range 1–3) for LTCF. 80% of reported outbreaks (n = 372) concerned HRMO and 20% (n = 91) were caused by other pathogens, mainly *C. difficile* and viruses (Additional file 2).

**Table 2:** Reason for reporting and severity of outbreaks reported to the SO-ZI/AMR (2012–2021), by healthcare setting and year

	2012^a^	2013	2014	2015	2016	2017	2018	2019	2020	2021	Total
**Hospitals**											
Total number of outbreaks	20	37	51	53	40	38	34	39	21	20	353
Number of outbreaks with request for support	3	8	4	4	0	0	0	0	1	1	21
**Reason for reporting (n (%))**											
Threat of ward closure	14 (70)	26 (70)	41 (80)	45 (85)	32 (80)	29 (76)	26 (76)	24 (62)	19 (90)	15 (75)	271 (77)
Ongoing transmission	5 (25)	8 (22)	8 (16)	4 (8)	3 (8)	4 (11)	2 (6)	2 (5)	2 (10)	2 (10)	40 (11)
Combination of both	0 (0)	0 (0)	0 (0)	0 (0)	4 (10)	1 (3)	2 (6)	2 (5)	0 (0)	1 (5)	10 (3)
Unknown	1 (5)	3 (8)	2 (4)	4 (8)	1 (3)	4 (11)	4 (12)	11 (28)	0 (0)	2 (10)	32 (9)
**Highest level severity phase** ^**b**^ **(n (%))**											
Phase 1	12 (60)	28 (76)	44 (86)	49 (92)	36 (90)	36 (95)	30 (88)	37 (95)	21 (100)	16 (80)	309 (88)
Phase 2	7 (35)	4 (11)	7 (14)	2 (4)	3 (8)	2 (5)	1 (3)	2 (5)	0 (0)	4 (20)	32 (9)
Phase 3	1 (5)	5 (14)	0 (0)	2 (4)	1 (3)	0 (0)	2 (6)	0 (0)	0 (0)	0 (0)	11 (3)
Phase 4	0 (0)	0 (0)	0 (0)	0 (0)	0 (0)	0 (0)	1 (3)	0 (0)	0 (0)	0 (0)	1 (0)
**Long-term care facilities**											
Total number of outbreaks	0﻿	2	4	8	10	22	24	20	13	7	110
Number of outbreaks with request for support	0	0	0	0	2	2	1	0	0	0	5
**Reason for reporting (n (%))**											
Threat of ward closure	0 (0)	1 (50)	3 (75)	8 (100)	7 (70)	6 (27)	5 (21)	4 (20)	4 (31)	4 (57)	42 (38)
Ongoing transmission	0 (0)	1 (50)	1 (25)	0 (0)	0 (0)	1 (5)	1 (4)	0 (0)	1 (8)	0 (0)	5 (5)
Combination of both	0 (0)	0 (0)	0 (0)	0 (0)	0 (0)	2 (9)	1 (4)	1 (5)	0 (0)	0 (0)	4 (4)
HRMO in healthcare facility other than hospital^c^	0 (0)	0 (0)	0 (0)	0 (0)	3 (30)	13 (59)	15 (63)	14 (70)	8 (62)	3 (43)	56 (51)
Unknown	0 (0)	0 (0)	0 (0)	0 (0)	0 (0)	0 (0)	2 (8)	1 (5)	0 (0)	0 (0)	3 (3)
**Highest level severity phase** ^**b**^ **(n (%))**											
Phase 1	0 (0)	2 (100)	4 (100)	8 (100)	9 (90)	18 (82)	22 (92)	20 (100)	13 (100)	7 (100)	103 (94)
Phase 2	0 (0)	0 (0)	0 (0)	0 (0)	1 (10)	3 (14)	2 (8)	0 (0)	0 (0)	0 (0)	6 (5)
Phase 3	0 (0)	0 (0)	0 (0)	0 (0)	0 (0)	1 (5)	0 (0)	0 (0)	0 (0)	0 (0)	1 (1)

SO-ZI/AMR: Healthcare-associated Infection and AntiMicrobial Resistance Monitoring Group.

^a^ The SO-ZI/AMR was initiated in April 2012

^b^ For definitions of outbreak phases, see Table 1

^c^ To stimulate long-term care facilities to report outbreaks and address them, they can apply for reimbursement of costs related to control measurements since 2018. At that time, this category was added as a reason for reporting, despite the fact that the other reasons may still apply

### Hospital outbreaks – characteristics over time

**Fig. 1:**
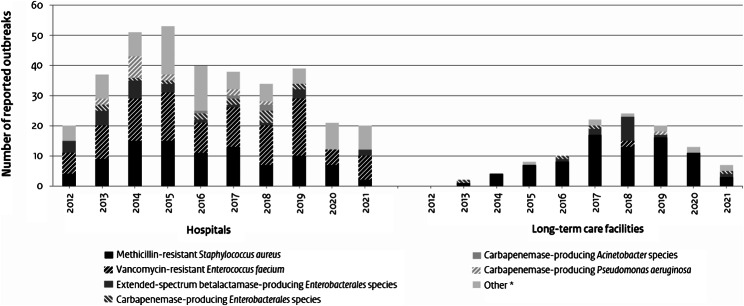
Distribution of (highly resistant) microorganisms among outbreaks reported to the SO-ZI/AMR (2012-2021^a^), by healthcare setting and year. SO-ZI/AMR: Healthcare-associated Infection and AntiMicrobial Resistance Monitoring Group. ^a^ April 2012 – December 2021. ^*^Clostridioides difficile, other *Enterobacterales*, methicillin-susceptible *S. aureus*, vancomycin-susceptible *E. faecium*, non-carbapenemase-producing *Acinetobacter* spp. / *P. aeruginosa*, viruses, other microorganisms

Among the 353 hospital outbreaks, (threat of) ward closure was the most common reason for reporting (271 outbreaks, 77%, Table 2). Support was requested by the hospital upon notification in 21 outbreaks (6%), the majority of which (n = 19) between 2012 and 2015. Outbreaks were mostly caused by vancomycin-resistant *Enterococcus faecium* (VRE; n = 116, 33%), MRSA (n = 93, 26%), and highly resistant *Enterobacterales* – either ESBL-producing, CPE, or FQAG-R – (n = 41, 12%, Fig. 1, Additional file 2). Highly resistant *Enterobacterales* were most often ESBL-producing (26 out of 41). A substantial number of outbreaks with *Pseudomonas aeruginosa* (n = 16), *Acinetobacter* species (n = 8), *C. difficile* (n = 15), norovirus (n = 28) and severe acute respiratory syndrome coronavirus 2 (SARS-CoV-2, n = 10) were reported. In total, 32 outbreaks (9%) involved carbapenemase-producing Gram-negative bacteria: 14 in *P. aeruginosa*, four in *Acinetobacter* species and 14 in *Enterobacterales* – of which ten *Klebsiella pneumoniae*, three *Enterobacter cloacae* and one *Citrobacter freundii*. Ten *Serratia* species outbreaks (non-highly resistant) were reported, mostly in neonatal (ICU) wards. The majority of outbreaks (n = 309, 88%) was controlled within 2 months and therefore had phase 1 as the highest level phase, whereas 9% (n = 32) progressed to phase 2 (duration longer than expected, Table 2). Eleven outbreaks (8 VRE, 1 MRSA and 2 *C. difficile*) reached phase 3, signifying a possible public health threat. One outbreak of carbapenemase-producing *C. freundii* was classified as phase 4, which indicates a (potential) insufficient effect of outbreak management response. The outbreak started in February 2018 and progressed to phase 4 in October 2018. It was mitigated to phase 3 in December 2018, and considered controlled (phase 0) in September 2019. No outbreaks progressed to phase 5.

### Hospital outbreaks – duration and size by microorganism (group)

Grouping outbreaks by microorganism, the duration until the outbreak was assigned phase 0 by the SO-ZI/AMR was longest for *E. faecium* (median 2.3, range 0.0-22.8 months, Table 3). In contrast, SARS-CoV-2 outbreaks were considered ongoing for only 1.0 month (range 0.4–1.4 months). Information on type(s) of department involved was available for 317/353 outbreaks (90%). In most of the *P. aeruginosa* outbreaks (11/13 with available information, 85%) an ICU ward was involved, whereas outbreaks with *S. aureus* involved ICU wards in only 19% (16/84 with available information). ICU wards were not involved in any of the outbreaks with viral pathogens. Data on outbreak size was available for 186/353 outbreaks (53%). Among these, outbreaks with SARS-CoV-2 affected the largest number of patients overall (median 15; range 0–34), however, the range of affected patients was largest for *E. faecium* outbreaks (median 9; range 2-483). Fourteen outbreaks involved more than 50 patients (10 *E. faecium*, 3 *C. difficile* and 1 norovirus). Outbreaks with CPE had the lowest number of affected patients (median 2; range 2–6), although information on the number of patients affected was only available for three CPE outbreaks.

**Table 3:** Duration, ICU involvement and size of outbreaks reported to the SO-ZI/AMR (2012-2021^a^), by healthcare setting and microorganism group

				Outbreak size			
	Number of outbreaks	Duration of outbreaks in months (median (range))^b^	Outbreaks on ICU wards (n (%))^c^	Number of outbreaks with information on patients involved	Number of patients involved per outbreak (median (range))^d^		
**Hospitals**							
*Staphylococcus aureus*	94	1.4 (0.2–18.5)	16/84 (19)	48	3 (1–18)		
*Enterococcus faecium*	117	2.3 (0.0–22.8)	40/104 (38)	64	9 (2–483)		
*Enterobacterales* species - ESBL	26	2.0 (0.2–13.8)	11/23 (48)	16	14 (4–27)		
*Enterobacterales* species - CPE	14	1.6 (0.3–18.6)	3/13 (23)	3	2 (2–6)		
*Enterobacterales* species - Other	12	1.4 (0.5–2.8)	4/11 (36)	8	6 (4–31)		
*Acinetobacter* species	8	2.0 (0.6–5.7)	3/7 (43)	4	5 (0–12)		
*Pseudomonas aeruginosa*	16	1.8 (0.9–3.5)	11/13 (85)	10	5 (2–50)		
*Clostridioides difficile*	15	2.1 (0.2–9.1)	5/15 (33)	9	8 (5–151)		
Norovirus	28	1.3 (0.5–2.0)	0/26 (0)	14	9 (2–74)		
SARS-CoV-2	10	1.0 (0.4–1.4)	0/10 (0)	10	15 (0–34)		
Other viruses^e^	7	1.1 (0.2–1.4)	0/6 (0)	5	7 (1–15)		
Other^f^	6	1.5 (1.1–5.5)	1/5 (20)	4	7 (3–14)		
**Long-term care facilities**							
*Staphylococcus aureus*	80	1.5 (0.0–8.9)	NA	35	4 (1–42)		
*Enterococcus faecium*	2	2.5 (2.3–2.7)	NA	0	NA		
*Enterobacterales* species - ESBL	13	1.9 (0.7–5.0)	NA	1	4 (4–4)		
*Enterobacterales* species - CPE	4	3.6 (2.4–8.9)	NA	2	4 (2–6)		
*Enterobacterales* species - Other	4	1.4 (0.9–8.7)	NA	2	6 (4–8)		
*Pseudomonas aeruginosa*	1	1.7 (1.7–1.7)	NA	0	NA		
Norovirus	2	1.1 (0.4–1.9)	NA	0	NA		
SARS-CoV-2	2	1.3 (1.2–1.3)	NA	2	16 (11–20)		
Other^g^	2	2.7 (0.0–5.5)	NA	1	16 (16–16)		

SO-ZI/AMR: Healthcare-associated Infection and AntiMicrobial Resistance Monitoring Group. NA: Not applicable

^a^ The SO-ZI/AMR was initiated in April 2012

^b^ Calculated as the time interval between either notification date or date of the monthly SO-ZI/AMR meeting in which the outbreak was first discussed (whichever came first), and the date of the SO-ZI/AMR meeting in which the outbreak was considered controlled (phase 0)

^c^ Information on the type(s) of department involved was not available for all outbreaks. The denominator presented reflects the number of outbreaks with available information

^d^ These numbers do not differentiate between infected and colonised patients. Staff members involved in the outbreak are not included

^e^ 1 astrovirus (2012), 2 measles virus (2013, 2014), 1 respiratory syncytial virus (2015), 1 enterovirus (2016), 1 rotavirus (2016), 1 influenza virus (2018)

^f^ 1 Bordetella pertussis (2015), 3 *Sarcoptes scabiei* (2015, 2018, 2021), 1 *Streptococcus pneumoniae* (2015), 1 *Candida norvegensis* (2017)

^g^ 2 *Sarcoptes scabiei* (2018, 2021)

### Long-term care facility outbreaks – characteristics over time

In five (5%) of the 110 outbreaks in LTCF, between 2016 and 2018, support was requested upon notification (Table 2). Most outbreaks concerned MRSA (n = 80, 72%) and highly resistant *Enterobacterales* (13 ESBL-producing (12%), four CPE (4%) and three FQAG-R (3%), Fig. 1, Additional file 2). VRE were involved in only 2 outbreaks (2%). Overall, the majority (n = 103, 94%) was controlled within 2 months (phase 1), six (5%) progressed to phase 2. One MRSA outbreak reached phase 3 (Table 2).

### Long-term care facility outbreaks – duration and size by microorganism (group)

Overall, *S. aureus* outbreaks were under discussion by the SO-ZI/AMR for a median of 1.5 months (range 0.0-8.9, Table 3). CPE outbreaks, although based on only four outbreaks, were considered ongoing for more than twice as long (median 3.6; range 2.4–8.9 months). SARS-CoV-2 outbreaks affected most patients (median 16; range 11–20), but the largest outbreak was an MRSA outbreak (42 patients).

## Discussion

The establishment of the SO-ZI/AMR in 2012 has resulted in a monthly transparent overview of nosocomial outbreaks in the Netherlands. Our results suggest that outbreaks with HRMO and other pathogens occurred regularly in Dutch healthcare settings in the past ten years, but that they mostly remained limited in size and were usually quickly controlled. The most frequently reported causative microorganisms were MRSA, VRE (in hospitals) and highly resistant *Enterobacterales*.

In the Netherlands, a combination of restrictive antibiotic use, high standards of hospital infection prevention and control, nationwide surveillance and an active MRSA search-and-destroy policy has proven to be able to limit MRSA prevalence and spread, as compared to other countries [13]. Accordingly, hospital MRSA outbreaks in our study were small and remained in phase 1/2. Carbapenemase-producing *Enterobacterales*, *P*. *aeruginosa* and *Acinetobacter baumannii* are considered as most feared HRMO since they can cause severe infections for which limited therapeutic options are available. Although the prevalence of CPE slowly increases in the Netherlands [6], the number of CPE outbreaks remains limited. The Dutch national bacterial pathogen surveillance showed that the CPE population is dynamic and diverse, suggesting that it is based on multiple introductions in the Netherlands rather than largescale within-country transmission [14]. In our data, outbreaks of carbapenemase-producing *K. pneumoniae* were more frequently reported than of carbapenemase-producing *E. coli*, which is in line with their increased ability of transmission [15]. In 2018, we noticed an increase in outbreaks caused by highly resistant *Enterobacterales*, and for the first time, an outbreak (NDM-producing *C. freundii*) was classified as phase 4. This outbreak was carefully monitored and the reporting hospital was offered support.

We compared our data with those from other countries. Norway, a country with similar resistance rates as the Netherlands [13], has implemented a web-based outbreak notification system, which is based on mandatory notification using criteria that differ from the SO-ZI/AMR, and is not limited to nosocomial outbreaks. They reported 157 nosocomial outbreaks in 2019, of which 17 (11%) were caused by highly resistant bacterial pathogens [16]. Although comparison with our data is not straightforward because of the differing notification criteria, this is considerably lower than the number of HRMO outbreaks we found in the same year (n = 52). However, taking into account the difference in the number of hospital and LTCF beds between Norway and the Netherlands (54,825 vs. 230,364, respectively, data from hospitals in 2019 and LTCF from 2012 [17–19]), the HRMO outbreak incidence is comparable with our data (31 vs. 23 per 100,000 beds in the respective countries). Germany reported 54 nosocomial outbreaks with HRMO bacteria between 1 November 2011 and 31 October 2012 [20]. However, the authors expect that many outbreaks were not reported during this pilot phase of the system, due to a narrow outbreak definition or lack of awareness on the hospital side. In our data, outbreaks of CPE, *P. aeruginosa*, and *Acinetobacter* species were rare and included few patients. In contrast, large outbreaks with these HRMO were reported in other countries in both Europe and the rest of the world, as confirmed in literature and in the Worldwide Outbreak Database, a database claiming to include all outbreak reports from medical literature in a standardised manner [21]. The moderate outbreak frequency in the Netherlands is probably associated with the low prevalence of these HRMO in the Netherlands and high standards of infection prevention which include the screening of high-risk patients upon hospital admittance (i.e. recent hospitalisation abroad). This allows healthcare facilities to take targeted control measures – e.g. isolation of all colonised or infected patients and exchange of information upon transfer between hospitals.

Since the establishment of the SO-ZI/AMR, valuable insights have been gained in the characteristics of outbreaks of (highly resistant) pathogens in Dutch healthcare settings, such as the severity, duration and causative agents. Furthermore, the SO-ZI/AMR contributes to an open communication which is essential in minimizing the public health threat of nosocomial outbreaks. Although the reporting to and monitoring by the SO-ZI/AMR may have contributed to increased awareness and thereby to controlling the outbreak in terms of limiting the duration or size of outbreaks, the true impact of the structure cannot be quantified with the available data. In 2017, an external qualitative evaluation of the SO-ZI/AMR was performed. As there were no outbreaks classified as phase 4/5 until 2017, the role of the SO-ZI/AMR in providing support and facilitating communication between healthcare facilities could not be evaluated and evaluation was focussed mainly on the signalling role of the SO-ZI/AMR. The central assessment of the severity of outbreaks by the SO-ZI/AMR as an authoritative body was perceived as one of the strengths of the structure. The fact that outbreaks in phase 1–3 are not followed by sanctions imposed by healthcare authorities, helps to normalise the fact that outbreaks in healthcare occur, which contributes to transparency and willingness to report and share information. The signalling function of the SO-ZI/AMR was also relevant when multiple outbreaks with NDM-producing *K. pneumoniae* and VIM-producing *P. aeruginosa* were noticed [22, 23]. The SO-ZI/AMR suggested the hospitals with an active outbreak of NDM-producing *K. pneumoniae* to share typing data and experience, including effectiveness of control measures, in order to help each other in further controlling the outbreaks. When multiple VIM-producing *P. aeruginosa* outbreaks occurred in one region of the country during a specific period, a meeting between the affected hospitals was initiated to investigate possible causes and infection control measures. The SO-ZI/AMR reported on the outcome of this meeting in their monthly bulletin, in order to inform other healthcare professionals. In these outbreaks, VIM-producing *P. aeruginosa* was suggested to be associated with previous use of antibiotics and persistence in environmental hospital sources [24–26]. Nationwide efforts are now undertaken to better understand these environmental sources and to contain these [27–30].

Our results also have limitations. First, our data may have suboptimal representativeness. Due to the voluntary nature of the SO-ZI/AMR and the narrow notification criteria, smaller outbreaks - that were quickly controlled and did not lead to potential ward closure – may not have been reported. One might argue that the likelihood of reporting depends on resource availability, IPC expertise, scientific interest, etc. During the period of the study, the overall median number of reported outbreaks reported per institute was 11 (range 3–23) for university hospitals, 3 (range 1–16) for non-university hospitals and 1 (range (1–3) for LTCF. However, these differences in numbers of reported outbreaks may also reflect actual differences in outbreak occurrence in these settings. There was no evidence of large HRMO outbreaks that were not reported to the SO-ZI/AMR and the perception is that the overview of reported HRMO outbreaks is fairly complete. Suboptimal representativeness was more likely for LTCF than for hospitals. The reason for adding LTCF in 2015 was to gain more insight in HRMO prevalence and control in LTCF. In 2016, a point prevalence study was undertaken on ESBL carriage in LTCF residents [31]. This increased the active detection and reporting of ESBL outbreaks. Still, outbreak detection, contact tracing and reporting of outbreaks remained limited in LTCF. However, once in 2018 a financial reimbursement rule was installed for outbreaks of HRMO in LTCF for which notification of the outbreak to the SO-ZI/AMR was required [32], reporting increased. Thus, especially in the earlier years, we have missed outbreaks in LTCF. Data reported during the COVID-19 pandemic (2020–2021) are also prone to suboptimal representativeness., as the number of outbreaks reported in 2020 and 2021 was lower compared to the years before. Underreporting (since hospitals faced overwhelming patient influx during the various COVID-19 waves) in these years could have contributed to this observation. For instance, the number of SARS-CoV-2 outbreaks in hospitals and particularly LTCF was likely much higher than reported to the SO-ZI/AMR, considering the number of LTCF locations with at least 1 positive test over time [33]. Still, also lower transmission rates due to increased infection prevention measures and reduced travel-related HRMO carriers, might be an explanation for the lower numbers of reported HRMO outbreaks during the COVID-19 pandemic. With regard to our results on non-HRMO pathogens (including viruses), it is important to note that these outbreaks generally do not meet the notification criteria. Some of them were reported anyway, and the SO-ZI/AMR did not discard these. Therefore our data do not provide a complete overview of outbreaks for all pathogens presented in this paper. A second limitation that should be accounted for when interpreting our results is the lack of accurate data on the start and end date of the outbreaks. We therefore defined outbreak duration as the duration between the date of reporting and the date of the SO-ZI/AMR monthly meeting in which the outbreak was assigned Phase 0. As notification of outbreaks to the SO-ZI/AMR was only indicated when continuity of care was jeopardised or transmission was ongoing despite control measures, it is likely that outbreaks were reported some time after they had started. Also, some outbreaks were only reported after they had been controlled. It is therefore likely that the duration of outbreaks in our results is an underestimation. As a third and final limitation that should be mentioned, the severity phase classification by the SO-ZI/AMR was a collective decision based on the expert panel’s professional judgement and strict criteria were not in place. Assigned classifications were not externally validated.

In 2022, procedural updates to the SO-ZI/AMR were implemented. Notification criteria were updated to also include an outbreak definition, the number of severity phases was reduced from five to three and include objective definitions, and outbreaks are now reported through a dedicated secured web-based platform using a personal account.

## Conclusions

The SO-ZI/AMR has become a solid monitoring body, essential to raise awareness of potential HRMO threats. It provided national insight into the characteristics – including severity based on expert consensus – of nosocomial outbreaks over the past decade. Our results suggest that HRMO outbreaks occurred regularly in Dutch healthcare settings (both hospitals and LTCF) but were usually controlled quickly. Although restricted therapeutic options are a problem at the patient level in case of HRMO infections, nosocomial HRMO outbreaks do not yet constitute a public health threat in the Netherlands.

### Electronic supplementary material

Below is the link to the electronic supplementary material.

Supplementary Material 1: Description of data: Additional file 1 provides examples of the baseline, follow-up and end-of-outbreak questionnaires used during the period of the study. These data are collected in order for the expert panel to (re)assess the potential public health risk of outbreaks.

Supplementary Material 2: Description of data: Additional file 2 provides the detailed distribution (n (%)) of microorganisms (including resistance profile if applicable) among outbreaks reported to the SO-ZI/AMR during the period of the study (2012-2021).

## Data Availability

The datasets generated and/or analysed during the current study are not publicly available due to legal agreements with notifying healthcare institutes but are available from the corresponding author on reasonable request.

## Abbreviations

COVID-19: Coronavirus disease 2019
CPE: carbapenemase-producing *Enterobacterales*
ESBL: extended-spectrum beta-lactamase
FQAG-R: fluoroquinolone and aminoglycoside-resistant
GGD: municipal health service in the Netherlands
HRMO: highly resistant microorganisms
ICU: intensive care unit
IGJ: Health and Youth Care inspectorate
LTCF: long-term care facilities
MRSA: methicillin-resistant *Staphylococcus aureus*
NDM: New Delhi metallo beta-lactamase
NethMap: national annual report on antimicrobial consumption and resistance in the Netherlands
NVMM: Dutch Society of Medical Microbiology
OXA: oxacillinase
RIVM-CIb: Center for Infectious Disease Control, National Institute for Public Health and the Environment
SARS-CoV-2: Severe acute respiratory syndrome coronavirus 2
SO-ZI/AMR: The Healthcare-associated Infections and AntiMicrobial Resistance Monitoring Group
VHIG: Association for Hygiene and Infection Control in the Netherlands
VIM: Verona Integron-encoded metallo beta-lactamase
VRE: vancomycin-resistant *Enterococcus faecium*
WIP: Dutch Working party on Infection Prevention
