# Supplementary material for: Description of a nationwide structure for monitoring nosocomial outbreaks of (highly resistant) microorganisms in the Netherlands: characteristics of outbreaks in 2012–2021
Source: Antimicrob Resist Infect Control. 2023 Dec 8;12:143. doi: 10.1186/s13756-023-01350-9 (PMC10709896; doi:10.1186/s13756-023-01350-9)
Supplement: Supplementary file 1 — Supplementary Material 1: Description of data: Additional file 1 provides examples of the baseline, follow-up and end-of-outbreak questionnaires used during the period of the study. These data are collected in order for the expert panel to (re)assess the potential public health risk of outbreaks. [file 13756_2023_1350_MOESM1_ESM.docx]

**Additional file 2**

Distribution of (highly resistant) microorganisms among outbreaks reported to the SO-ZI/AMR by healthcare setting and year

**Contents:**

- **Table A2a. Distribution (n (%)) of (highly resistant) microorganisms among hospital outbreaks reported to the SO-ZI/AMR (2012-2021), by year**
- **Table A2b. Distribution (n (%)) of (highly resistant) microorganisms among long-term care facility outbreaks reported to the SO-ZI/AMR (2012-2021), by year**

**Table A2a. Distribution (n (%)) of (highly resistant) microorganisms among hospital outbreaks reported to the SO-ZI/AMR (2012-2021), by year**

|  | **2012^a^** | **2013** | **2014** | **2015** | **2016** | **2017** | **2018** | **2019** | **2020** | **2021** | **Total** |
| --- | --- | --- | --- | --- | --- | --- | --- | --- | --- | --- | --- |
| Total number of outbreaks | 20 | 37 | 51 | 53 | 40 | 38 | 34 | 39 | 21 | 20 | 353 |
| **Microorganism - resistance profile** | | | | | | | | | | | |
| *Staphylococcus aureus* - MRSA | 4 (20) | 9 (24) | 15 (29) | 15 (28) | 11 (28) | 13 (34) | 7 (21) | 10 (26) | 7 (33) | 2 (10) | 93 (26) |
| *Staphylococcus aureus* - MSSA | 0 (0) | 0 (0) | 0 (0) | 0 (0) | 1 (3) | 0 (0) | 0 (0) | 0 (0) | 0 (0) | 0 (0) | 1 (0) |
| *Enterococcus faecium* - VRE | 7 (35) | 11 (30) | 14 (27) | 16 (30) | 10 (25) | 13 (34) | 13 (38) | 19 (49) | 5 (24) | 8 (40) | 116 (33) |
| *Enterococcus faecium* - Non-VRE | 1 (5) | 0 (0) | 0 (0) | 0 (0) | 0 (0) | 0 (0) | 0 (0) | 0 (0) | 0 (0) | 0 (0) | 1 (0) |
| *Enterobacterales* species - ESBL^b^ | 4 (20) | 5 (14) | 6 (12) | 3 (6) | 1 (3) | 1 (3) | 1 (3) | 3 (8) | 0 (0) | 2 (10) | 26 (7) |
| *Enterobacterales* species - CPE^c^ | 0 (0) | 2 (5) | 1 (2) | 1 (2) | 2 (5) | 2 (5) | 4 (12) | 2 (5) | 0 (0) | 0 (0) | 14 (4) |
| *Enterobacterales* species - FQAG-R^d^ | 0 (0) | 0 (0) | 0 (0) | 0 (0) | 1 (3) | 0 (0) | 0 (0) | 0 (0) | 0 (0) | 0 (0) | 1 (0) |
| *Enterobacterales* species - Serratia species | 0 (0) | 0 (0) | 1 (2) | 1 (2) | 3 (8) | 0 (0) | 0 (0) | 2 (5) | 2 (10) | 1 (5) | 10 (3) |
| *Enterobacterales* species - Non-HRMO^e^ | 0 (0) | 1 (3) | 0 (0) | 0 (0) | 0 (0) | 0 (0) | 0 (0) | 0 (0) | 0 (0) | 0 (0) | 1 (0) |
| *Acinetobacter* species - CPA^f^ | 0 (0) | 0 (0) | 0 (0) | 0 (0) | 1 (3) | 1 (3) | 2 (6) | 0 (0) | 0 (0) | 0 (0) | 4 (1) |
| *Acinetobacter* species - FQAG-R^g^ | 0 (0) | 0 (0) | 1 (2) | 0 (0) | 0 (0) | 0 (0) | 0 (0) | 0 (0) | 0 (0) | 0 (0) | 1 (0) |
| *Acinetobacter* species - Non-HRMO^h^ | 0 (0) | 0 (0) | 0 (0) | 0 (0) | 1 (3) | 0 (0) | 1 (3) | 0 (0) | 0 (0) | 1 (5) | 3 (1) |
| *Pseudomonas aeruginosa* - CPPA | 0 (0) | 2 (5) | 7 (14) | 2 (4) | 0 (0) | 2 (5) | 1 (3) | 0 (0) | 0 (0) | 0 (0) | 14 (4) |
| *Pseudomonas aeruginosa* - Non-HRMO | 0 (0) | 0 (0) | 0 (0) | 2 (4) | 0 (0) | 0 (0) | 0 (0) | 0 (0) | 0 (0) | 0 (0) | 2 (1) |
| *Clostridioides difficile* | 3 (15) | 5 (14) | 1 (2) | 2 (4) | 1 (3) | 1 (3) | 0 (0) | 1 (3) | 1 (5) | 0 (0) | 15 (4) |
| Norovirus | 0 (0) | 1 (3) | 4 (8) | 7 (13) | 6 (15) | 4 (11) | 3 (9) | 2 (5) | 0 (0) | 1 (5) | 28 (8) |
| SARS-CoV-2 | 0 (0) | 0 (0) | 0 (0) | 0 (0) | 0 (0) | 0 (0) | 0 (0) | 0 (0) | 6 (29) | 4 (20) | 10 (3) |
| Other viruses^i^ | 1 (5) | 1 (3) | 1 (2) | 1 (2) | 2 (5) | 0 (0) | 1 (3) | 0 (0) | 0 (0) | 0 (0) | 7 (2) |
| Other^j^ | 0 (0) | 0 (0) | 0 (0) | 3 (6) | 0 (0) | 1 (3) | 1 (3) | 0 (0) | 0 (0) | 1 (5) | 6 (2) |

SO-ZI/AMR: Healthcare-associated Infection and AntiMicrobial Resistance Monitoring Group. MRSA: methicillin-resistant *Staphylococcus aureus*, MSSA: methicillin-susceptible *Staphylococcus aureus*, VRE: vancomycin-resistant *Enterococcus faecium*, ESBL: extended-spectrum beta-lactamase, CPE: carbapenemase-producing *Enterobacterales* species, FQAG-R: fluoroquinolone and aminoglycoside resistant, HRMO: highly resistant microorganism, CPA: carbapenemase-producing *Acinetobacter* species, CPPA: carbapenemase-producing *Pseudomonas aeruginosa*, SARS-CoV-2: Severe acute respiratory syndrome coronavirus 2.

^a^ The SO-ZI/AMR was initiated in April 2012.

^b^ 15 *Klebsiella pneumoniae* (3 in 2012, 4 in 2013, 1 in 2014, 2 in 2015, 1 in 2017, 1 in 2018, 2 in 2019, 1 in 2021), 6 *Enterobacter cloacae* (1 in 2012, 3 in 2014, 1 in 2019, 1 in 2021), 3 *Escherichia coli* (2 in 2014, 1 in 2016), 2 *Citrobacter freundii* (1 in 2013, 1 in 2015).

^c^ 10 *Klebsiella pneumoniae* (2 in 2013, 1 in 2015, 1 in 2016, 1 in 2017, 3 in 2018, 2 in 2019), 3 *Enterobacter cloacae* (1 in 2014, 1 in 2016, 1 in 2017), 1 *Citrobacter freundii* (2018).

^d^ 1 *Escherichia coli* (2016).

^e^ 1 *Enterobacter cloacae* (2013).

^f^ 2 *Acinetobacter baumannii* complex (2016, 2018), 2 *Acinetobacter*, species unknown (2017, 2018).

^g^ 1 *Acinetobacter baumannii* (2014).

^h^ 2 *Acinetobacter baumannii* complex (2016, 2021), 1 *Acinetobacter*, species unknown (2018).

^i^ 1 astrovirus (2012), 2 measles virus (2013, 2014), 1 respiratory syncytial virus (2015), 1 enterovirus (2016), 1 rotavirus (2016), 1 influenza virus (2018).

^j^ 1 *Bordetella pertussis* (2015), 3 *Sarcoptes scabiei* (2015, 2018, 2021), 1 *Streptococcus pneumoniae* (2015), 1 *Candida norvegensis* (2017).

**Table A2b. Distribution (n (%)) of (highly resistant) microorganisms among long-term care facility outbreaks reported to the SO-ZI/AMR (2012-2021), by year**

|  | **2012^a^** | **2013** | **2014** | **2015** | **2016** | **2017** | **2018** | **2019** | **2020** | **2021** | **Total** |
| --- | --- | --- | --- | --- | --- | --- | --- | --- | --- | --- | --- |
| Total number of outbreaks | 0 | 2 | 4 | 8 | 10 | 22 | 24 | 20 | 13 | 7 | 110 |
| **Microorganism - resistance profile** | | | | | | | | | | | |
| *Staphylococcus aureus* - MRSA | 0 (0) | 1 (50) | 4 (100) | 7 (88) | 8 (80) | 17 (77) | 13 (54) | 16 (80) | 11 (85) | 3 (43) | 80 (73) |
| *Enterococcus faecium* - VRE | 0 (0) | 0 (0) | 0 (0) | 0 (0) | 0 (0) | 0 (0) | 2 (8) | 0 (0) | 0 (0) | 0 (0) | 2 (2) |
| *Enterobacterales* species - ESBL^b^ | 0 (0) | 0 (0) | 0 (0) | 0 (0) | 1 (10) | 2 (9) | 8 (33) | 1 (5) | 0 (0) | 1 (14) | 13 (12) |
| *Enterobacterales* species - CPE^c^ | 0 (0) | 1 (50) | 0 (0) | 0 (0) | 1 (10) | 1 (5) | 0 (0) | 0 (0) | 0 (0) | 1 (14) | 4 (4) |
| *Enterobacterales* species - FQAG-R^d^ | 0 (0) | 0 (0) | 0 (0) | 1 (13) | 0 (0) | 1 (5) | 0 (0) | 0 (0) | 1 (8) | 0 (0) | 3 (3) |
| *Enterobacterales* species - Non-HRMO^e^ | 0 (0) | 0 (0) | 0 (0) | 0 (0) | 0 (0) | 1 (5) | 0 (0) | 0 (0) | 0 (0) | 0 (0) | 1 (1) |
| *Pseudomonas aeruginosa* - CPPA | 0 (0) | 0 (0) | 0 (0) | 0 (0) | 0 (0) | 0 (0) | 0 (0) | 1 (5) | 0 (0) | 0 (0) | 1 (1) |
| Norovirus | 0 (0) | 0 (0) | 0 (0) | 0 (0) | 0 (0) | 0 (0) | 0 (0) | 2 (10) | 0 (0) | 0 (0) | 2 (2) |
| SARS-CoV-2 | 0 (0) | 0 (0) | 0 (0) | 0 (0) | 0 (0) | 0 (0) | 0 (0) | 0 (0) | 1 (8) | 1 (14) | 2 (2) |
| Other^f^ | 0 (0) | 0 (0) | 0 (0) | 0 (0) | 0 (0) | 0 (0) | 1 (4) | 0 (0) | 0 (0) | 1 (14) | 2 (2) |

SO-ZI/AMR: Healthcare-associated Infection and AntiMicrobial Resistance Monitoring Group. MRSA: methicillin-resistant *Staphylococcus aureus*, VRE: vancomycin-resistant *Enterococcus faecium*, ESBL: extended-spectrum beta-lactamase, CPE: carbapenemase-producing *Enterobacterales*, FQAG-R: fluoroquinolone and aminoglycoside resistant, HRMO: highly resistant microorganism, CPA: carbapenemase-producing *Acinetobacter* species, CPPA: carbapenemase-producing *Pseudomonas aeruginosa*, SARS-CoV-2: Severe acute respiratory syndrome coronavirus 2.

^a^ The SO-ZI/AMR was initiated in April 2012.

^b^ 12 *Escherichia coli* (2 in 2017, 8 in 2018, 1 in 2019, 1 in 2021), 1 *Klebsiella pneumoniae* (2016).

^c^ 2 *Klebsiella pneumoniae* (2013, 2016), 1 *Escherichia coli* (2017). One outbreak involved both *Citrobacter freundii* and *Klebsiella variicola*.

^d^ 3 *Escherichia coli* (2015, 2017, 2020).

^e^ 1 *Salmonella kentucky* (2017).

^f^ 2 *Sarcoptes scabiei* (2018, 2021).
