# Supplementary material for: Description of a nationwide structure for monitoring nosocomial outbreaks of (highly resistant) microorganisms in the Netherlands: characteristics of outbreaks in 2012–2021
Source: Antimicrob Resist Infect Control. 2023 Dec 8;12:143. doi: 10.1186/s13756-023-01350-9 (PMC10709896; doi:10.1186/s13756-023-01350-9)
Supplement: Supplementary file 2 — Supplementary Material 2: Description of data: Additional file 2 provides the detailed distribution (n (%)) of microorganisms (including resistance profile if applicable) among outbreaks reported to the SO-ZI/AMR during the period of the study (2012-2021). [file 13756_2023_1350_MOESM2_ESM.docx]

**Additional file 1:** SO-ZI/AMR questionnaires used for outbreak data collection

**Contents:**

- **1a Baseline questionnaire**
- **1b Follow-up questionnaire**
- **1c End-of-outbreak questionnaire**

During the period of the study, these questionnaires were slightly adapted a few times. The questionnaires included in this additional file are a combination of several versions. They therefore serve as an illustration of the questionnaires.

The questionnaires were translated from their original language (Dutch) by SHS Woudt.

## 1a Baseline questionnaire

This questionnaire was required to be filled out upon notification.

**Date notification*:** dd-mm-yy

**Notifier (Name)*:**

**Initials:**

**Phone number*:**

**Email*:**

**Profession notifier*:**  Medical microbiologist, infection control practitioner, elderly care physician, other

**Healthcare facility (Name and location)*:**

**Type of healthcare facility*:** Hospital, Long-term care facility, Other (if other, provide details)

**Is the healthcare facility part of a health care organization?** Yes/No (If yes, provide details)

**Place (city/village) healthcare facility location:**

**Place (province) healthcare facility location:**

**Contact person healthcare facility** (when other than notifier):

**Phone number contact person healthcare facility** (when other than notifier):

**Laboratory where cultures are performed** (when other than notifying laboratory):

**Place (city) of laboratory where cultures are performed** (when other than notifying laboratory):

**Reason for reporting***

1. Outbreak threatens healthcare continuity, for instance when wards need to be closed or when a healthcare institute stops new admissions to control the outbreak
2. Transmission of the outbreak strain continues despite appropriate infection control measures
3. Combination of 1 and 2
4. None of the above, outbreak concerns a HRMO in a facility other than a hospital

All outbreaks in hospitals meeting criterion 1 and/or 2, should be reported to the SO-ZI/AMR.

For other healthcare facilities, notification is limited to HRMO, but criteria 1 and/or 2 are not required to be met.

**Identity disclosed on password-protected part of NVMM website*?** Yes/No

Do you agree to disclosing the name and location of the healthcare facility for your fellow medical microbiologists, infection control practitioners, elderly care physicians?

**Identity disclosed in the monthly SO-ZI/AMR report (part of the Weekly Overview of The Early Warning Meeting of Infectious Diseases)**: Yes/No

Do you agree to disclosing the name and location of the healthcare facility for professionals subscribed to receiving this overview? These professionals include medical microbiologists, infection control practitioners, infectious disease specialists, medical doctors working for the Municipal Health Service, other medical doctors working for Community Health Services, elderly care physicians, policy workers from the national Centre for Infectious Disease Control and the government.

**Short outbreak description*:**

Please provide a short outbreak description, for publication in the monthly SO-ZI/AMR report as part of the Weekly overview of The Early Warning Meeting of Infectious Diseases. This description could include: name/place/region of healthcare facility, microorganism (and resistance profile if applicable), possible source of the index patient/resident, possible cause of transmission, degree of transmission (e.g. number of patients, staff, wards), measures taken, degree of outbreak containment, microorganism typing results, etc.).

**Microorganism*:** (drop-down list)

*1| Enterococcus faecium*

*2| Staphylococcus aureus*

*3| Escherichia coli*

*4| Klebsiella pneumoniae*

*5| Pseudomonas aeruginosa*

*6| Norovirus*

*7| Klebsiella oxytoca*

*8| Clostridium difficile*

*9| Serratia spp.*

*10| Enterobacter spp.*

*11| Acinetobacter spp.*

*12| Stenotrophomonas maltophilia*

*13| Streptococcus pneumoniae*

*14| Rotavirus*

*15| Other*

**If Other, provide details:**

**Type of HRMO, according to guidelines published by** the Dutch Working party on Infection Prevention***:** (multiple answers possible)

- esbl|ESBL (extended-spectrum B-lactamase) production
- mrsa|MRSA (methicillin-resistant *S. aureus*)
- vre|VRE (amoxicillin- and vancomycin-resistant *Enterococcus* spp.)
- cp|CP (carbapenemase production)
- cr|CR (carbapenem-resistant without evidence of carbapenemase production or a carbapenemase gene
- FQAG|Fluoroquinolone- and aminoglycoside-resistant *Enterobacterales***/*Acinetobacter* spp.
- MRPseu|Multi-resistant *P. aeruginosa* (resistance to ≥3 antimicrobial groups among carbapenems, aminoglycosides, fluoroquinolones, ceftazidime, piperacillin)
- Steno|Co-trimoxazole (trimethoprim-sulfamethoxazole resistance in *Stenotrophomonas maltophilia*
- prp|PRP (Penicillin- or vancomycin-resistant *Streptococcus pneumoniae*
- Other|Other (if other, please specify)
- NA|not applicable

***Enterobacterales* include: *Escherichia coli*, *Klebsiella pneumoniae*, *Klebsiella oxytoca*, *Serratia* spp*.*, *Providencia* spp,. *Enterobacter* spp., *Proteus* spp., *Morganella morganii*, *Citrobacter* spp.

Resistance mechanism/gene, if applicable:

- Not applicable
- *mecA*
- *mecC*
- *vanA*
- *vanB*
- VIM
- IMP
- OXA-48
- Other OXA(-like) gene
- NDM
- KPC
- Other (if other, please specify)

**Results molecular typing** (if applicable):

**Was the index person admitted to a hospital abroad for >24 hours in the past 2 months?*** Yes/No/Unknown

**Has the index person been transferred from another healthcare facility? If yes, which one?***

Yes/No/Unknown

**Total number of patients/residents with the outbreak microorganism (infected and/or colonized) on date of notification:**

**Total number of staff with the outbreak microorganism on date of notification:**

**Number of wards with infected/colonized patients/residents on date of notification:**

**Type of ward(s) where outbreak is ongoing** (multiple answers possible)**:**

- Hospital – ICU
- Hospital – Internal medicine
- Hospital – Surgery
- Hospital – Paediatrics
- Hospital – Neonatology
- LTCF – Psychogeriatric ward
- LTCF – Somatic ward
- LTCF – Combined somatic/psychogeriatric ward
- LTCF – Rehabilitation ward
- Other (if other, please specify)

**Date of last confirmed case:** dd-mm-yy

**Date outbreak terminated** (if applicable)**:** dd-mm-yy

**Did transmission occur from the healthcare facility to elsewhere (on date of notification)?**

- Unknown
- Yes, to the community
- Yes, to other healthcare facility/facilities
- No

**Prevention/control measures installed (on date of notification):** (multiple answers possible)

- Contact tracing
- Targeted/periodic screening of high-risk patients and/or those with high length of stay
- Contact isolation precautions
- Investigation of source/environment (e.g. surfaces, sinks)
- Closing of ward(s)
- Set up outbreak management team
- Education of staff
- Performing audits on wards
- Enhanced cleaning and disinfection
- Separate toilet / sanitary tools for infected/colonized patients/residents
- Cohort isolation of infected/colonized patients/residents on another location
- Other (if other, please specify)

**Would you like to request extra/external help or information?** If yes, please specify

Yes/No

**Additional comments:**

**Is this notification related to an outbreak that was reported before? If yes, please specify which one**

Yes/No

## 1b Follow-up questionnaire

This questionnaire was sent to notifiers of active outbreaks approximately 10 days before the SO-ZI/AMR expert panel’s monthly meeting, and was requested to be returned no later than 1 day before the meeting.

**Do you consider the outbreak terminated (for hospitals) or under control (for LTCF)?*** Yes/No

**In case the outbreak is still ongoing, please provide the following (approximate) information:**

- Number of suspected cases
- Number of confirmed cases
- Wards that are involved
- In case contact tracing is/was performed: number of patients that are being screened, and the percentage of patients that has already been screened
- Specific measures taken (other than reported previously)
- Frequency of ward screening rounds
- For large outbreaks (e.g. VRE with >20 patients or ongoing for >3 months): an epidemic curve displaying the new and total number of patients over time

## 1c End-of-outbreak questionnaire

This questionnaire was sent to notifiers of outbreaks that were classified as Phase 0 (outbreak controlled) by the SO-ZI/AMR expert panel during their last monthly meeting (based on information provided through the follow-up questionnaire). It was requested to be returned within 2 weeks.

Based on the information you provided, the SO-ZI/AMR decided to classify the outbreak you reported as Phase 0 (outbreak controlled). To complete the comprehensive view on this outbreak, we kindly request you to return the following questionnaire.

If at any time transmission with the same microorganism is detected again, or continuity of care is (or could become) jeopardized, please report the outbreak again via the NVMM website.

**Healthcare facility (Name, location)*:**

**Place (city/village) healthcare facility location:**

**Place (province) healthcare facility location:**

**Date of notification to the SO-ZI/AMR** (available on NVMM website or in confirmation email)

**Microorganism:**

**Total number of patients/residents with the outbreak microorganism (infected and/or colonized):**

**Total number of staff with the outbreak microorganism (infected and/or colonized):**

**Probable outbreak start date (as determined after the outbreak was controlled):** dd-mm-yy

**Date of last confirmed case:** dd-mm-yy

**Was this case detected via retrograde screening:** Yes/No

**Implementation date of last update of infection control measures:** dd-mm-yy

*The following questions are optional*

**Number of wards where transmission has likely occurred:**

**Type of ward(s) with infected/colonized patients** (multiple answers possible)**:**

- Hospital – ICU
- Hospital – Internal medicine
- Hospital – Surgery
- Hospital – Paediatrics
- Hospital – Neonatology
- LTCF – Psychogeriatric ward
- LTCF – Somatic ward
- LTCF – Combined somatic/psychogeriatric ward
- LTCF – Rehabilitation ward
- Other (if other, please specify)

**Prevention/control measures installed (on date of notification):** (multiple answers possible)

- Contact tracing
- Targeted/periodic screening of high-risk patients and/or those with high length of stay
- Contact isolation precautions
- Investigation of source/environment (e.g. surfaces, sinks)
- Closing of ward(s)
- Set up outbreak management team
- Education of staff
- Performing audits on wards
- Enhanced cleaning and disinfection
- Separate toilet / sanitary tools for infected/colonized patients/residents
- Cohort isolation of infected/colonized patients/residents on another location
- Other (if other, please specify)

**Probable source or hypothesis on the cause of transmission:**

**Did transmission occur from the healthcare facility to elsewhere (on date of notification)?**

- Unknown
- Yes, to the community
- Yes, to other healthcare facility/facilities
- No

**Please provide (as attachment) an epidemic curve of the outbreak (optional).**
